# Incorporation of Testicular Ultrasonography and Hair Steroid Concentrations in Bull Breeding Soundness Evaluation

**DOI:** 10.3390/vetsci10060373

**Published:** 2023-05-25

**Authors:** Alessio Cotticelli, Luigi Navas, Alfonso Calabria, Giovanna Bifulco, Giuseppe Campanile, Tanja Peric, Alberto Prandi, Michael J. D’Occhio, Marco Russo

**Affiliations:** 1 Department of Veterinary Medicine and Animal Production, Federico II University, 80137 Naples, Italy; alessio.cotticelli@unina.it (A.C.); luigi.navas@unina.it (L.N.); calabria_123@hotmail.it (A.C.); giovanna.bifulco@unina.it (G.B.); giucampa@unina.it (G.C.); marco.russo@unina.it (M.R.); 2 Department of Agricultural, Environmental and Animal Science, University of Udine, 33100 Udine, Italy; alberto.prandi@uniud.it; 3 Faculty of Agriculture and Environment, The University of Sydney, Sydney, NSW 2006, Australia; michael.docchio@sydney.edu.au

**Keywords:** testicular ultrasonography, hair steroids, semen, bull

## Abstract

**Simple Summary:**

Bulls’ subfertility has a major impact on the efficiency of production and profitability of cattle enterprises. Bulls typically undergo a bull breeding soundness evaluation (BBSE) to predict potential fertility. The present study investigated if a more comprehensive index of indicative fertility could be developed in bulls by including testicular ultrasonography and hormonal status in the BBSE. Bulls with homogeneous testicular parenchyma showed a higher percentage of motile sperm post-thawing compared with bulls with heterogeneous parenchyma. In bulls with homogenous parenchyma, the percentage of motile sperm, progressively motile sperm, and motility yield were positively correlated with hair DHEA-S concentration. The use of testicular ultrasonography and DHEA-S status in the BBSE would provide a more comprehensive assessment of potential fertility in bulls. In addition, ultrasonography can be used in the BBSE when the evaluation of semen parameters is not available.

**Abstract:**

Testicular ultrasonography and steroid concentrations (cortisol, dehydroepiandrosterone sulfate (DHEA-S), cortisol/DHEA-S ratio, testosterone) in hair were examined for their utility in the bull breeding soundness evaluation (BBSE). Beef and dairy bulls (*n* = 16; 2.7 ± 0.4 years old; body condition score 3.2 ± 0.1) of five breeds were maintained under the same conditions at an accredited semen collection center. Bulls underwent routine semen collection twice weekly for 12 weeks and semen was processed and cryopreserved. Ultrasonography and hair sampling were undertaken at the last semen collection. Bulls with homogeneous testicular parenchyma (*n* = 8) had a higher (*p* < 0.05) percentage of motile sperm post-thawing compared with bulls with heterogeneous parenchyma (*n* = 8). There were no differences (*p* > 0.05) in the hair concentrations of cortisol, DHEA-S, and testosterone between bulls with homogeneous and heterogeneous parenchyma. In bulls with homogeneous parenchyma, hair DHEA-S concentration was positively correlated with percentage motile sperm (R^2^ = 0.76), progressively motile sperm (R^2^ = 0.70), and motility yield (R^2^ = 0.71). The findings indicate that the integration of testicular ultrasonography and hair DHEA-S status in the BBSE could provide a more comprehensive assessment of indicative fertility in bulls. Additionally, ultrasonography can be used in the BBSE when the evaluation of semen parameters is not available.

## 1. Introduction

Bulls with low fertility have a major negative impact on the efficiency of production and profitability of cattle enterprises [1,2]. Hence, bulls routinely undergo a bull breeding soundness evaluation (BBSE) before they are used for natural or assisted breeding. BBSE involves an assessment of overall structural soundness, integrity of the reproductive organs, and semen quality [1]. Noninvasive testicular ultrasonography has undergone preliminary investigation as an additional parameter for inclusion in the BBSE. Ultrasonography provides information on the integrity of testicular parenchyma, and the relationship to spermatogenesis [3,4]. The homogeneity of testicular parenchyma, as judged by ultrasonography, was reported to have an important bearing on spermatogenesis and fertility in males. In men, testicular inhomogeneity, characterized by the presence of fibrotic tissue on ultrasound, was associated with impaired sperm quality and azoospermia [5]. The relationship between testicular homogeneity and sperm production and fertility is less clear for bulls. In an early study, there were no differences in sperm abnormalities between bulls with fibrotic foci in testicular parenchyma and bulls without fibrotic foci [6]. Subsequent studies also reported no clear association between the integrity of testicular parenchyma and semen quality in bulls [7,8,9]. However, in a study with a small number of bulls, testicular lesions were associated with a low BBSE score and poor semen quality [10].

Spermatogenesis is influenced by many factors including metabolic and endocrine status [11]. The brain–adrenal axis is involved in metabolic homeostasis [12] and it also influences the brain–gonadal axis [13,14]. Adrenal glucocorticoids, including cortisol, typically have a negative impact on testicular function including spermatogenesis [15,16]. Glucocorticoids are elevated during stress, and chronic stress can be associated with impaired sperm production [17]. The adrenals also secrete the androgens dehydroepiandrosterone (DHEA) and DHEA sulphate (DHEA-S) [18,19]. In cattle, DHEA and DHEA-S are suppressed when cortisol is elevated during chronic stress [20]. The inverse relationship between cortisol and DHEA/DHEA-S led to the proposal that DHEA and DHEA-S could be antagonistic to cortisol [20].

Allostasis is a term used to describe mechanisms whereby the body adapts to stressors to maintain healthy homeostasis. Allostatic load is the build-up of stressors over time and the impact on the brain and somatic tissues. In cattle and other species, the amount of cortisol present in hair is reflective of the short- to medium-term activity of the brain–adrenal axis and provides an index of allostatic load [21,22]. Hair and blood concentrations of DHEA and DHEA-S are also reflective of allostatic load in cattle [22]. The effect of cortisol and DHEA-S on semen parameters and fertility has not been thoroughly investigated in bulls. The present study investigated the effects of testicular ultrasonography and hair steroids (cortisol, DHEA-S, cortisol/DHEA-S ratio, testosterone) on semen parameters in bulls. The aim was to determine the association between testicular ultrasonography and hair steroids with semen parameters in bulls. If ultrasonography and hair steroids were shown to be related to semen parameters, they could be used in BBSE if semen assessment was not available. The hypothesis tested was that the integrity of testicular parenchyma is related to semen parameters in bulls. The accurate selection of bulls for fertility is particularly important when bulls of high commercial value are used extensively in assisted breeding programs.

## 2. Materials and Methods

All experimental procedures complied with the Italian legislation on animal care (Legislative Decree n. 116, 27/1/1992). The study had approval from the Ethical Animal Care and Use Committee of the University of Naples Federico II (Protocol PG72021/0130477).

### 2.1. Animals

The study involved sixteen bulls (2.7 ± 0.4 years old, body condition score 3.2 ± 0.1) of five breeds: Pezzata rossa italiana (*n* = 7), Holstein Friesian (*n* = 5), Limousine (*n* = 2), Charolaise (*n* = 1), and Chianina (*n* = 1). Animals were maintained under the same management at an accredited National Semen Collection Center. The study lasted 12 weeks and testicular ultrasound examination and hair sampling were undertaken at the same time as the last semen collection, according to the retrospective value provided by the hair matrix.

### 2.2. Testicular Ultrasonography

For testicular ultrasound examination, bulls were restrained in a bovine steel stanchion. The scrotal skin was cleaned and ultrasonographic gel was applied to increase the quality of the ultrasound image. A B-mode ultrasound scanner (MyLab™AlphaVET- Esaote S.p.a, Genova, Italy) equipped with a 13–3 MHz linear array probe was used to image the testes of each bull; the same settings were used for focus, gain, brightness, and contrast, standardized at the machine median settings. The ultrasound transducer was held vertically (parallel to the long axis of the testes) on the caudal surface of the scrotum. The image was aligned until the mediastinum of the testes was clear and apparent [7]. The image was then frozen and saved. This process was repeated with the ultrasound transducer in the horizontal plane (at the widest part of the testis) and both views were repeated for the other testis. A validated scoring system was used to identify bulls with a homogeneous testicular parenchyma and bulls with a heterogeneous parenchyma [23,24]. In brief, the scoring system adopts a six-point scale with scores of 0–5 encompassing normal homogenous patterns of echotexture to very severe fibrosis throughout the testis [8]. All images were obtained by the same operator.

### 2.3. Sample Collection and Processing

#### 2.3.1. Hair

The hair in cattle grows at approximately 0.6–1.0 cm per month and animals show a full molt approximately every 3 months [25]. The concentration of steroids in hair therefore provides an integrated measure of secretion during the preceding 2 to 3 months [25,26,27]. The integrated value avoids the short-term and diurnal variations in steroid secretion and is a more accurate indicator of the prevailing steroidal status of animals. Hair samples can be readily obtained and processed compared with blood samples. Hence, hair steroid concentrations were used in the present study. Hair was obtained from the scapular region of bulls using a razorblade and cut close to the skin at the same time as the last semen collection. Samples represented the integrated steroid concentration over the 12-week duration of the study. Samples were stored in dry tubes at room temperature and in the dark until analysis.

#### 2.3.2. Semen

Bulls underwent semen collection twice weekly for 12 weeks as part of the routine commercial activity of the authorized National Semen Collection Center. Bulls were trained to serve an artificial vagina (IMV, L’Aigle, France). A total of 384 ejaculates were collected during the study.

### 2.4. Semen Evaluation

Semen was assessed and processed immediately after collection. The volume of semen was estimated by weighing the ejaculates within 5 min of collection and incubation at 35 °C. Initial assessments on fresh semen included the following: ejaculate volume (mL), motility (% motile spermatozoa), and total concentration (10^6^ sperm/mL) [28,29]. Semen concentration and motility were determined by the computer-assisted semen analysis system (CASA, Sperm Vision, Minitube GmbH, Tiefenbach, Germany). The technical settings used in the present study were the following: depth of sample chamber 20 µm, light adjustment 100–155, temperature of analysis 37 °C, dimension of sperm heads 22–99 µm^2^, frame rate 60 s^−1^, immotile AOC < 3.5 µms^−1^, lateral motile DSL < 15 µms^−1^, VSL < 20 µms^−1^ and VAP < 24.9 µms^−1^, progressively motile STR > 0.5 and LIN > 0.35, non-linear STR < 0.5 and LIN < 0.35, curvilinear DAP/Radius = 3 and LIN < 0.5, round pat area average < 40 µm^2^, BCF = 0, and AOC > 8 [30]. Semen samples were diluted to a final concentration of 40 to 80 × 10^6^ spermatozoa/mL with egg yolk-based freezing extenders (Tryladil, Minitube GmbH, Tiefenbach, Germany) before packaging into 0.25 mL straws. Straws containing extended semen were then incubated at 5 °C overnight. Freezing was initiated by transferring the straws into a fixed temperature freezing chamber (Minitube GmbH, Tiefenbach, Germany) at −140 °C for 15 min, and subsequently plunging them into liquid nitrogen. Assessments on frozen-thawed semen were performed within 5 min after thawing and included the percentage of motile sperm (% motile), percentage of progressively motile sperm (% sperm PM), percentage of viable sperm (% live sperms), and motility yield (proportion of sperm migrating into the medium).

### 2.5. Hair Steroid Assays

Hair samples were prepared for the steroid assay as previously described [25]. Briefly, the hair samples were washed in isopropanol (Sigma-Aldrich, St. Louis, MO, USA), and approximately 60 mg of trimmed hair was extracted with methanol (Sigma-Aldrich, St. Louis, MO) for 16 h. Vials were then evaporated to dryness at 37 °C under an airstream suction hood and the remaining residue was dissolved in 0.35 mL of phosphate-buffered saline (PBS), 0.05 M, pH 7.5.

Cortisol [25,26], DHEA-S [25], and testosterone [27] concentrations were measured using solid-phase microtiter radioimmunoassay assays. Cortisol was measured using a commercial rabbit anti-cortisol antibody (Analitical Antibodies-Bologna, Italy) with cross-reactivities: cortisol 100%; cortisone 4.3%; corticosterone 2.8%; 11-deoxycorticosterone 0.7%; 17-hydroxyprogesterone 0.6%; dexamethasone 0.1%; progesterone < 0.01%; 17-hydroxypregnenolone < 0.01%; DHEA-S < 0.01%; androsterone sulfate < 0.01%; pregnenolone < 0.01%. DHEA-S was measured using a commercial rabbit anti-dehydroepiandrosterone sulfate-7ß-CM-BSA antibody (SpiBio, Montigny le Bretonneux, France) with cross-reactivities: DHEA-S 100%; 4-Androsten-3,17-dione (4-androstenedione) 0.2%; 4-Androsten-17-ol-3-one (testosterone) < 0.01%; 5-Androsten-3-ol-17-one (dehydroepiandrosterone, DHEA) < 0.01%; 5-Androstan-3-ol-17-one (androsterone) < 0.01%. Testosterone was measured using a commercial anti-testosterone-3-carboxymethyloxime-BSA antibody (Analytical Antibodies-Bologna, Italy) with cross-reactivities: testosterone 100%; 5α-dihydrotestosterone 43.2%; 5α-androstanedione 33.1%; 5β-androstanedione 11.4%; 5α-androstan-3a,17b-diol 9.4%; androstenedione 0.4%; progesterone, DHEA, oestradiol 0.01%; cortisol < 0.001%. For cortisol, intra- and inter-assay coefficients of variation (CV) were 3.6 and 9.8%, respectively, and the sensitivity was 24.6 pg/mL (Riasmart software, Perkin-Elmer Life Sciences, Boston, MA, USA). For DHEA-S, the intra- and inter-assay coefficients of variation were 3.6% and 12.7%, respectively, and the sensitivity was 15.8 pg/mL. For testosterone, the intra- and inter-assay CV were 3.5% and 12.8%, respectively, and the sensitivity was 17.6 pg/mL.

### 2.6. Statistical Analyses

Statistical analyses were carried out using SPSS (28.0) for Windows 10 (SPSS Inc., Chicago, IL, USA). The initial dataset was edited, discriminating both for missing information and outliers (values lying 3 standard deviations below/above the mean). The number of samples excluded was the same between the homogenous and heterogenous bulls, which were characterized by similar coefficients of dispersion. The final dataset consisted of 236 ejaculates (14.7 ± 1.4/bull). The normal distribution of all data was confirmed using the Shapiro–Wilk test. Bulls were used as the experimental units. Multivariate analysis of variance (general linear model) was used to compare hair steroids of bulls (dependent variables); testicular parenchyma and breed were the fixed factors, and their interaction was also considered. Data on semen characteristics were analyzed by ANOVA for repeated measures with testicular parenchyma (homogenous/heterogenous) as the main factor, and breed and cortisol as random. The day of collection was the repeated measure. Multiple linear regression was performed (forward stepwise procedures) with steroid concentrations as independent variables, and fertility parameters (mean values) as dependents. Potential independent and dependent variables were first tested for potential correlations using Pearson correlations and only significant correlations (*p* < 0.05) were included in the regression model. Pearson correlation was also used to exclude possible intercorrelations between the independent variables. Unless otherwise stated, the results are presented as mean ± standard error and significance was set at *p* < 0.05.

## 3. Results

### 3.1. Testicular Ultrasonography

**Figure 1:**
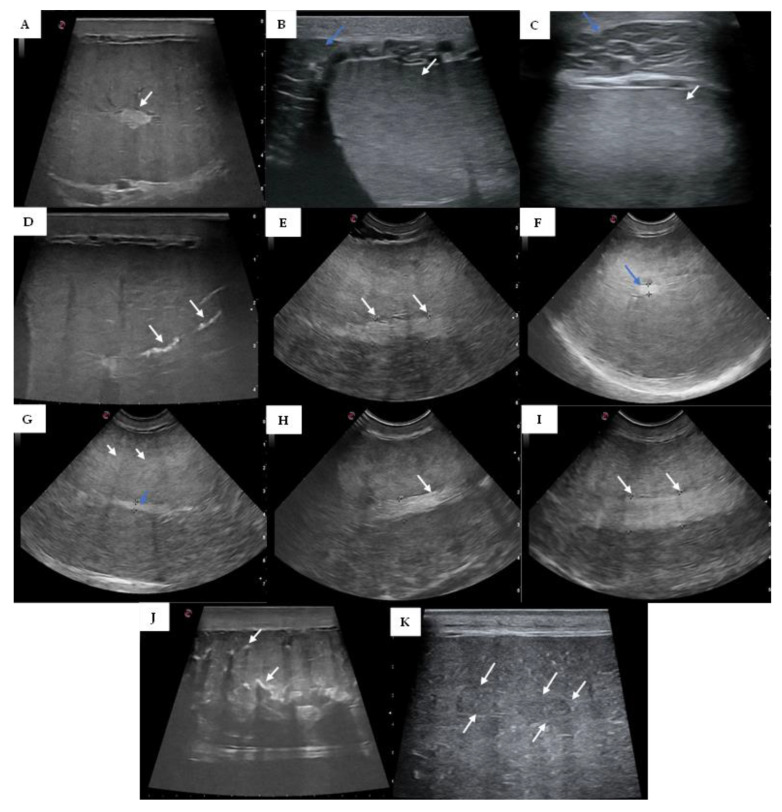
Representative ultrasound images for bulls with homogeneous testicular parenchyma (**A**–**C**): (**A**) medium echogenicity with the mediastinum testis as a central or slightly eccentric hyperechoic focus (white arrow); (**B**) homogenous testicular parenchyma (white arrow) and head of the epididymis (blue arrow); (**C**) homogenous testicular parenchyma (white arrow) and body of the epididymis (blue arrow); and bulls with heterogeneous testicular parenchyma (**D**–**K**): (**D**) linear hyperechoic, non-shadowing foci (white arrows) filling the rete tubules indicative of spermiostasis; (**E**) slight heterogeneous testicular parenchyma and distension of mediastinum testis (8.7 and 8.8 mm left and white arrow respectively); (**F**) slight heterogeneous testicular parenchyma and mediastinum testis of 4.2 mm (blue arrow); (**G**) slight heterogeneous testicular parenchyma with small hyperechoic foci (white arrows) and mediastinum testis of 3.6 mm (blue arrow); (**H**) heterogeneous testicular parenchyma, distension, and thickening of mediastinum testis (6.8 mm, white arrows); (**I**) bilateral testicular hypotropia and severe mediastinum distension (10.4 and 10.8 mm left and white arrow respectively); (**J**) coarse echotexture with multiple hyperechoic foci (white arrows) with distal acoustic enhancement scattered throughout the parenchyma; (**K**) inhomogeneous echotexture with hypoechoic areas scattered throughout the parenchyma.

Eight bulls had homogeneous testicular parenchyma (Holstein Friesian, 4; Pezzata rossa italiana, 2; Limousine, 1; Charolais, 1) (fibrosis score 0–2) (Figure 1). For the other eight bulls, four bulls had slight to mild generalized heterogeneous parenchyma (Pezzata rossa italiana, 2; Holstein Friesian, 1; Limousine, 1), three bulls had clearly noticeable hyperechoic foci of calcification (Pezzata rossa italiana, 2; Chianina, 1), and one bull had severe hydrocele (Pezzata rossa italiana) (score 3–5) (Figure 1).

### 3.2. Testicular Parenchyma and Spermatozoan Parameters

Fertility parameters of fresh semen did not differ between homogenous and heterogenous bulls (Table 1).

Bulls with homogeneous parenchyma had a higher (*p* < 0.05) percentage of motile sperm post-thawing (Table 2).

### 3.3. Steroid Concentrations

Concentrations in hair of cortisol, DHEA-S, and testosterone, and the cortisol/DHEA-S ratio, are shown in Table 3. For all three steroids, there were no significant differences between bulls with homogeneous or heterogeneous testicular parenchyma.

### 3.4. Steroid Concentrations and Semen Parameters

The relationships between hair DHEA-S concentration and motile sperm (%), progressively motile sperm (%), and motility yield for bulls with homogeneous testicular parenchyma are shown in Table 4. Hair DHEA-S was positively related to motile sperm (R^2^ = 0.76), progressively motile sperm (R^2^ = 0.70), and motility yield (R^2^ = 0.71).

## 4. Discussion

The present study examined whether the incorporation of testicular ultrasonography and hair steroid concentrations in the bull breeding soundness evaluation (BBSE) would provide a broader and more comprehensive index of indicative fertility. Another objective was to determine whether testicular ultrasonography could be used in the BBSE when semen evaluation is not available. Bulls with homogeneous testicular parenchyma had a higher percentage of motile sperm post-thawing compared with bulls with heterogeneous parenchyma. This finding could be interpreted to suggest that the sperm of bulls with homogenous parenchyma has a higher tolerance to cryopreservation and thawing compared with the sperm of bulls with heterogeneous parenchyma. This was an important observation as sperm motility is related to fertility in bulls [28,29]. Ultrasonography represents a practical, non-invasive procedure and adds important information to the BBSE. In an earlier study, the condition of the parenchyma was reported to be predictive of semen quality 2 to 4 weeks after ultrasound examination in bulls [7].

There were no differences in hair concentrations of testosterone, cortisol, and DHEA-S between bulls with homogeneous or heterogeneous testicular parenchyma. Previous studies in cattle and other species have reported an inverse relationship between cortisol and DHEA-S, and it was suggested that DHEA-S may partly counterbalance the negative impact of elevated cortisol on physiological and endocrine functions [20,22,31,32]. The cortisol/DHEA-S ratio was also considered an index of allostatic load [22]. The finding on the cortisol/DHEA-S ratio in the present study was interpreted to indicate that bulls with homogeneous and heterogeneous testicular parenchyma experienced the same allostatic load and did not have compromised endocrine function. This could be expected as all bulls experienced the same handling and management at an accredited National Semen Collection Center. Therefore, factors other than cortisol, and the cortisol/DHEA-S ratio, contributed to differences in testicular parenchyma condition in the present study. In this regard, testicular status was reported to have a genetic component [33].

Percentage motile sperm, progressively motile sperm, and motility yield were positively correlated with hair DHEA-S concentration in bulls with homogeneous parenchyma. This relationship may have been partly due to the prohormonal role of DHEA-S and its conversion to androgens and/or estrogens in peripheral target tissues [34]. As noted above, sperm motility and morphology are closely correlated with fertility [29]. Hair DHEA-S concentrations reflect adrenal secretion and assimilation in hair during the preceding weeks, and give a longer-term integration of DHEA-S status. Ultrasonography is now used routinely for monitoring reproductive function in females, and hair sampling is used for genomic testing in males and females. Hair sampling is more practical than blood for hormonal and genetic evaluation.

As noted, there are conflicting reports on relationships between testicular parenchyma and testis hormonal and spermatogenic function in bulls. The present study has provided strong evidence that the condition of the parenchyma is reflective of spermatogenesis. Given the practical implementation of ultrasonography and hair sampling, the case can be made for inclusion in the BBSE, or ultrasonography can be used when semen evaluation is not available.

## 5. Conclusions

The present study has shown that bulls with homogeneous testicular parenchyma have sperm with a greater resilience to cryopreservation than the sperm of bulls with heterogeneous testicular parenchyma. This is an important finding as bulls of high commercial value are used extensively in artificial insemination. The study also highlighted a positive relationship between hair DHEA-S and important sperm fertility parameters. A limitation of the present study was the relatively small number of bulls tested and the absence of the BBSE. Notwithstanding, it could be concluded that the inclusion of testicular ultrasonography and hair DHEA-S in the standard BBSE is practical and would provide a more integrated and comprehensive assessment of fertility in bulls. Finally, ultrasonography can be used when the evaluation of semen parameters is not available.

## Figures and Tables

**Table 1:** Fertility parameters for fresh semen in bulls with homogeneous (*n* = 8) and heterogeneous (*n* = 8) testicular parenchyma. Results are mean ± sem.

	Testicular Parenchyma		
Fertility Parameters (Fresh Semen)	Homogenous	Heterogenous	*p*
Ejaculate volume	6.7 ± 0.5	6.6 ± 0.5	0.86
Sperm concentration (×10^6^/mL)	710 ± 133	940 ± 186	0.32
Motile sperm (%)	54.5 ± 8.2	60.5 ± 9.6	0.64

**Table 2:** Fertility parameters post-thawing in bulls with homogeneous (*n* = 8) and heterogeneous (*n* = 8) testicular parenchyma. Results are mean ± sem. ^a,b^ *p* < 0.05.

	Testicular Parenchyma		
Fertility Parameters (Post-Thawing)	Homogenous	Heterogenous	*p*
Motile sperm (%)	65.7 ± 4.7 ^a^	51.6 ± 3.8 ^b^	0.02
Progressively motile sperm (%)	41.3 ± 4.5	33.8 ± 3.9	0.21
Viable sperm (%)	49.1 ± 4.5	44.2 ± 4.2	0.44
Motility yield (%)	41.9 ± 2.4	41.8 ± 2.5	0.97

**Table 3:** Hair steroid concentrations (pg/mg) for bulls with homogenous testicular parenchyma and bulls with heterogenous testicular parenchyma. HCC, hair cortisol concentration; HTC, hair testosterone concentration; HDC, hair DHEA-S concentration; C/DHEA-S ratio (×100). Results are estimated marginal mean ± sem.

	Testicular Parenchyma		
Hair Steroid Concentrations	Homogenous	Heterogenous	*p*
HCC	1.1 ± 0.1	1.3 ± 0.1	0.21
HDC	6.7 ± 0.7	7.8 ± 0.6	0.54
HTC	4.8 ± 0.6	4.2 ± 0.6	0.53
C/DHEA-S ratio	17.6 ± 1.8	16.6 ± 1.9	0.70

**Table 4:** Relationship between hair dehydroepiandrosterone sulphate (DHEA-S) and fertility parameters in bulls with homogeneous testicular parenchyma.

Fertility Parameter		*p*
Motile sperm (%)	22.88 + 2.88 (DHEA-S); R^2^ = 0.76	0.003
Progressively motile sperm	8.53 + 2.66 (DHEA-S); R^2^ = 0.70	0.006
Motility yield	17.19 + 2.86 (DHEA-S); R^2^ = 0.71	0.006

## Data Availability

Data are available upon reasonable request to the corresponding author.

## References

[1] Barth A.D. (2018). Review: The Use of Bull Breeding Soundness Evaluation to Identify Subfertile and Infertile Bulls. Animal.

[2] Butler M.L., Bormann J.M., Weaber R.L., Grieger D.M., Rolf M.M. (2020). Selection for Bull Fertility: A Review. Transl. Anim. Sci..

[3] Abdelnaby E.A. (2022). Testicular Haemodynamics, Plasma Testosterone and Oestradiol Concentrations, and Serum Nitric Oxide Levels in the Egyptian Buffalo Bull after a Single Administration of Human Chorionic Gonadotropin. Reprod. Domest. Anim..

[4] Fadl A.M., Abdelnaby E.A., El-Sherbiny H.R. (2022). Supplemental Dietary Zinc Sulphate and Folic Acid Combination Improves Testicular Volume and Haemodynamics, Testosterone Levels and Semen Quality in Rams under Heat Stress Conditions. Reprod. Domest. Anim..

[5] Spaggiari G., Granata A.R.M., Santi D. (2020). Testicular Ultrasound Inhomogeneity Is an Informative Parameter for Fertility Evaluation. Asian J. Androl..

[6] Eilts B.E., Pechman R.D. (1988). B-mode ultrasound observations of bull testes during breeding soundness examinations. Theriogenology.

[7] Arteaga A.A., Barth A.D., Brito L.F.C. (2005). Relationship between Semen Quality and Pixel-Intensity of Testicular Ultrasonograms after Scrotal Insulation in Beef Bulls. Theriogenology.

[8] Barth A.D., Alisio L., Avilés M., Arteaga A.A., Campbell J.R., Hendrick S.H. (2008). Fibrotic Lesions in the Testis of Bulls and Relationship to Semen Quality. Anim. Reprod. Sci..

[9] Tomlinson M., Jennings A., Macrae A., Truyers I. (2017). The Value of Trans-Scrotal Ultrasonography at Bull Breeding Soundness Evaluation (BBSE): The Relationship between Testicular Parenchymal Pixel Intensity and Semen Quality. Theriogenology.

[10] Yimer N., Haron A.W. (2011). Trans-Scrotal Ultrasonography and Breeding Soundness Evaluation of Bulls in a Herd of Dairy and Beef Cattle with Poor Reproductive Performance. Artic. Pertanika J. Trop. Agric. Sci..

[11] Brito L.F.C. (2021). Endocrine Control of Testicular Development and Initiation of Spermatogenesis in Bulls. Bovine Reproduction.

[12] Mormède P., Andanson S., Aupérin B., Beerda B., Guémené D., Malmkvist J., Manteca X., Manteuffel G., Prunet P., van Reenen C.G. (2007). Exploration of the Hypothalamic-Pituitary-Adrenal Function as a Tool to Evaluate Animal Welfare. Physiol. Behav..

[13] Brkovich A.M., Fisher W.A. (1998). Psychological Distress and Infertility: Forty Years of Research. J. Psychosom. Obstet. Gynaecol..

[14] Hardy M.P., Gao H.B., Dong Q., Ge R., Wang Q., Chai W.R., Feng X., Sottas C. (2005). Stress Hormone and Male Reproductive Function. Cell Tissue Res..

[15] Barth A.D., Bowman P.A. (1994). The Sequential Appearance of Sperm Abnormalities after Scrotal Insulation or Dexamethasone Treatment in Bulls. Can. Vet. J..

[16] Thibier M., Rolland O. (1976). The effect of dexamethasone (DXM) on circulating testosterone (T) and luteinizing hormone (LH) in young postpubertal bulls. Theriogenology.

[17] Whirledge S., Cidlowski J.A. (2013). A Role for Glucocorticoids in Stress-Impaired Reproduction: Beyond the Hypothalamus and Pituitary. Endocrinology.

[18] Gabai G., Mongillo P., Giaretta E., Marinelli L. (2020). Do Dehydroepiandrosterone (DHEA) and Its Sulfate (DHEAS) Play a Role in the Stress Response in Domestic Animals?. Front. Vet. Sci..

[19] Whitham J.C., Bryant J.L., Miller L.J. (2020). Beyond Glucocorticoids: Integrating Dehydroepiandrosterone (DHEA) into Animal Welfare Research. Animals.

[20] Almeida P.E., Weber P.S.D., Burton J.L., Zanella A.J. (2008). Depressed DHEA and Increased Sickness Response Behaviors in Lame Dairy Cows with Inflammatory Foot Lesions. Domest. Anim. Endocrinol..

[21] Meyer J.S., Novak M.A. (2012). Minireview: Hair Cortisol: A Novel Biomarker of Hypothalamic-Pituitary- Adrenocortical Activity. Endocrinology.

[22] Peric T., Corazzin M., Romanzin A., Bovolenta S., Prandi A., Montillo M., Comin A. (2017). Cortisol and DHEA Concentrations in the Hair of Dairy Cows Managed Indoor or on Pasture. Livest. Sci..

[23] Chapwanya A., Callanan J., Larkin H., Keenan L., Vaughan L. (2008). Breeding soundness evaluation of bulls by semen analysis, testicular fine needle aspiration cytology and trans-scrotal ultrasonography. Ir. Vet. J..

[24] Chenoweth P.J., McPherson F.J. (2016). Bull Breeding Soundness, Semen Evaluation and Cattle Productivity. Anim. Reprod. Sci..

[25] Probo M., Peric T., Fusi J., Prandi A., Faustini M., Veronesi M.C. (2021). Hair Cortisol and Dehydroepiandrosterone Sulfate Concentrations in Healthy Beef Calves from Birth to 6 Months of Age. Theriogenology.

[26] Peric T., Comin A., Corazzin M., Montillo M., Cappa A., Campanile G., Prandi A. (2013). Short Communication: Hair Cortisol Concentrations in Holstein-Friesian and Crossbreed F1 Heifers. J. Dairy Sci..

[27] Stradaioli G., Peric T., Montillo M., Comin A., Corazzin M., Veronesi M.C., Prandi A. (2017). Hair Cortisol and Testosterone Concentrations and Semen Production of Bos Taurus Bulls. Ital. J. Anim. Sci..

[28] Barceló-Fimbres M., Campos-Chillón L.F., Seidel J.E. (2011). In Vitro Fertilization Using Non-Sexed and Sexed Bovine Sperm: Sperm Concentration, Sorter Pressure, and Bull Effects. Reprod. Domest. Anim..

[29] Seidel G.E. (2012). Several Insights on Evaluation of Semen. Anim. Reprod..

[30] O’Meara C., Henrotte E., Kupisiewicz K., Latour C., Broekhuijse M., Camus A., Gavin-Plagne L., Sellem E. (2022). The Effect of Adjusting Settings within a Computer-Assisted Sperm Analysis (CASA) System on Bovine Sperm Motility and Morphology Results. Anim. Reprod..

[31] Morgan C.A., Southwick S., Hazlett G., Rasmusson A., Hoyt G., Zimolo Z., Charney D. (2004). Relationships Among Plasma Dehydroepiandrosterone Sulfate and Cortisol Levels, Symptoms of Dissociation, and Objective Performance in Humans Exposed to Acute Stress. Arch. Gen. Psychiatry.

[32] Saczawa M.E., Graber J.A., Brooks-Gunn J., Warren M.P. (2013). Methodological Considerations in Use of the Cortisol/DHEA(S) Ratio in Adolescent Populations. Psychoneuroendocrinology.

[33] de Fonseca P.A.S., dos Santos F.C., Lam S., Suárez-Vega A., Miglior F., Schenkel F.S., de Diniz L.A.F., Id-Lahoucine S., Carvalho M.R.S., Cánovas A. (2018). Genetic Mechanisms Underlying Spermatic and Testicular Traits within and among Cattle Breeds: Systematic Review and Prioritization of GWAS Results. J. Anim. Sci..

[34] Labrie F. (2015). All Sex Steroids Are Made Intracellularly in Peripheral Tissues by the Mechanisms of Intracrinology after Menopause. J. Steroid Biochem. Mol. Biol..

